# Treatment of epilepsy – towards precision

**DOI:** 10.12688/f1000research.16448.1

**Published:** 2018-12-13

**Authors:** John Paul Leach

**Affiliations:** 1 University of Glasgow, Wolfson Medical School Building, Glasgow, Lanarkshire, G12 8QQ, UK

**Keywords:** Epilepsy, Genetics, Treatment, Safety

## Abstract

Epilepsy was among the first disease areas to begin to apply principles of precision medicine to its treatment. This review looks at the role of investigation in ensuring the safety and effectiveness of antiepileptic drug treatment. Using sound principles, we can see that the use of genetic testing will advance treatment of epilepsy in reducing harm and adverse effects and enhancing efficacy.

## Introduction

Epilepsy is one of the most common neurological conditions, affecting around 0.5% to 1% of any given population. It presents a serious health burden, has a recognised (but low) mortality rate, and accounts for 2% to 3% of admissions to acute general medical services.

The outlook for newly diagnosed epilepsy remains good for most people. Around 65% to 70% of patients will attain long-term seizure freedom with the first or second antiepileptic drug (AED) tried ^1^. In everyday practice, the choice of initial AED on diagnosis currently depends mostly on syndromic classification, the treatment choice being framed by whether the epilepsy is deemed to be genetic generalised or focal in onset ^2^. This crude separation of the epilepsies depends on the clinical and sometimes electroencephalography features present at the time of diagnosis.

Publications from the Scottish Intercollegiate Guidelines Network (SIGN 143) ^3^ and the National Institute for Health and Clinical Excellence ^4^ have provided clear guidance on many aspects of epilepsy care and have helped guide initial choice of medication and the treatment of refractory epilepsy. After failure of the first drugs, the physician can move to subsequent monotherapy or additional polypharmacy, the choice being largely determined by the likelihood of particular side effects, presence (or otherwise) of concomitant medications, and the presence of or potential for other health conditions in the patient with epilepsy ^5^.

Despite the arrival of almost 20 new drugs for epilepsy, the rate of seizure freedom has remained largely unchanged across the decades ^6^, and although tolerability may have improved in that time ^7^, it becomes clear that we will need to continue to develop more sophisticated ways to determine choice of medication in patients with new-onset or refractory epilepsy.

Some of the motivation for improving specificity and predictability of treatment outcomes becomes clearer with the emergent story of long-term sequelae to exposure to sodium valproate *in utero*. While the biological basis for this adverse effect remains to be clarified, it is vital that the epilepsy community and the regulatory bodies come together to safeguard and promote the use of one of our most effective therapies for genetic generalised epilepsy. Precision medicine has the potential to make epilepsy treatment safer and more effective and it falls to us to ensure that this happens.

## Precision medicine and epilepsy

### Improving tolerability

Idiosyncratic drug reactions to AEDs are serious but uncommon. One of the most serious complications of carbamazepine (CBZ) use is Stevens–Johnson syndrome, a life-threatening condition that has a mortality rate of around 50% and that develops in around 21 cases per 100,000 patient exposed to CBZ ^8^. This idiosyncratic reaction has been shown to be related in some populations to specific HLA markers ^9^: HLA-B*1502 is an immunological marker found mainly in patients of Southeast Asian descent. Its presence is associated in this population with a fourfold increase in the risk of CBZ-associated rash and a 70-fold increase in the risk of Stevens–Johnson syndrome. Such a specific stratification of risk has influenced practice in some patient groups ^10^ and such work has given us to the ability to target CBZ and phenytoin use in specific populations of Southeast Asian descent only to those patients in whom it is safe ^10^, thereby avoiding increasing risk of harm in patients with newly diagnosed or refractory epilepsy. Such associations are not as firmly correlated for other drugs or in other racial subtypes. The presence of the HLA-A*301 haplotype in those of Northern European descent increases the risk of any form of CBZ-related skin reaction from 5% to 26% ^11^. Calls have been made to promote the cost-effectiveness of screening for this haplotype before CBZ is prescribed in these populations ^12^.

While such work has helped predict the rarer and serious drug side effects, large ongoing studies of AED use in newly diagnosed epilepsy incorporate genetic testing to try to find genetic associations with less serious but more common side effects of AED use, such as mood disorder, tremor, weight gain, or cognitive change. The mathematical and technical challenges therein are still significant, not least of which will be the clear delineation and definition of these adverse effects and the need to recruit exceptionally large cohorts.

### Improving effectiveness

Currently, the choice of treatment in patients with newly diagnosed epilepsy depends on some fairly basic clinical characterisation. The SANAD (Standard and New Antiepileptic Drug) studies from 2007 ^7, 13^ showed the benefits of this, and the two treatment arms were determined by the then-contemporary clinical classification of partial or generalised epilepsy. The choice of treatments was different in each arm in this randomised open study and helped provide a context for drug preference in newly diagnosed epilepsy. In focal epilepsy, the optimal seizure responses were seen with lamotrigine and CBZ, and the former demonstrated somewhat improved tolerability.

The choice of AED for genetic generalised epilepsy has always been more limited since the traditional sodium channel blockers can be associated with an increase in seizure frequency and severity. In the generalised arm of the SANAD study ^13^, sodium valproate, rather than topiramate and lamotrigine, was most effective at inducing seizure freedom. However, the deleterious effect of this drug in pregnancy has led to a continued search for safe and efficacious treatment which does not present pregnancy-related problems. The SANAD2 study looks to compare levetiracetam and sodium valproate in patients with newly diagnosed genetic generalised epilepsy and is due to report in 2019.

Given the crude clinical factors that currently influence initial treatment choice, it should be time for principles of precision medicine to come to the fore in our decision making in many stages of epilepsy care. Thankfully this day seems to be closer ^14^. Genetic studies are becoming increasingly common in patients with epilepsy.

The discovery of a specifically treatable substrate is rare but is potentially life-saving and life-changing, most commonly noted when there is a sodium channel mutation (SCN1A) causing Dravet syndrome or a genetic Glut-1 transporter deficiency (SCL2A1). Such a discovery will firmly place stiripentol ^15^ or a ketogenic diet ^16^, respectively, early in the treatment pathway.

The discovery of specific genetic mutations has helped us to repurpose drugs with specific actions which may have been used in entirely unrelated conditions ^17^. Delineation of a specific channelopathy has allowed us to predict efficacy of quinidine for its antiepileptic effect in patients with epilepsy associated with KCNT1 mutations ^18^. It may be hoped that further analysis of genetic variations in those with Dravet syndrome will highlight those with serotonergic changes most likely to benefit from new drugs such as the amphetamine derivative fenfluramine, justifying the associated cardiovascular risk ^19^.

The suggested promise of screening for polymorphisms in p-glycoprotein as a marker or predictor of drug resistance has not been fulfilled in the longer term ^20^, but we should retain hopes of more substantial breakthrough in coming years.

Even where there is no specific targeted treatment currently available, the ability to counsel patients fully or even just provide more insight to affected families will have a markedly positive effect in improving the journey for patients and families ^21^.

In adult clinics, genetic analysis in patients with epilepsy is becoming increasingly important, most especially in those patients with associated learning difficulties or progressive encephalopathy or in those with refractory generalised epilepsy. Lindy *et al*. ^22^ reported a positive yield from next-generation sequencing or copy number variation analysis in 15% of patients in a panel of tests looking at up to 70 genes.

Even in early epilepsy, before treatment has been followed up in the long term, genetic testing may shed light on the underlying pathophysiology; around 9% of children with complex febrile convulsions have genetic changes in SCN1A testing ^23^.

More widespread testing in adult patients can enhance diagnostic yield further. Whole exome sequencing provides a specific diagnosis in 12.5% in patients with non-lesional focal epilepsy with a positive family history ^24^.

Although any patient with refractory epilepsy may benefit from genetic screening, such testing will be of most importance in patients with early-onset seizures (less than 3 years of age), a family history of seizures, associated neurological deficit, or learning disability ^25^. In short, clinical features suggesting widespread neuronal dysfunction are more likely to have an underlying genetic abnormality uncovered by today’s testing.

## Conclusions

For most of our patients the key to improving outcome remains in the careful electroclinical assessment, allowing full classification and allocation of the most appropriate treatment to invoke the highest chances of seizure freedom and our best attempts at prognostication. Where epilepsy is refractory to treatment or where there is other significant developmental or neurological difficulties, we require careful use of genetic and metabolic testing to fully implement the principles of precision medicine. These principles remain an ideal for other disorders but are becoming a reality for patients with epilepsy. We know that, as genetic technologies advance, this will become an increasingly important part of management, not only for our patients with refractory or complex epilepsy but even in those whose epilepsy is newly diagnosed. In such uncertain times, we know that our epilepsy clinics will require us to be able to relate to complex genetics as they unfold but more importantly to be able to relate to our patients and their families. As they progress though their life journey with its challenges, they need a clinician with the knowledge, experience, and wisdom to interpret the genetic findings.
